# *Morganella morganii* and *Enterococcus faecalis* endophthalmitis following intravitreal injection

**DOI:** 10.1186/s12886-023-03198-4

**Published:** 2023-11-10

**Authors:** Michael Kvopka, WengOnn Chan, Duleepa Baranage, David Sia

**Affiliations:** 1 Department of Ophthalmology, The Royal Adelaide Hospital, Port Road, 5000 Adelaide, SA Australia; 2 Discipline of Ophthalmology and Visual Sciences, University of Adelaide, Adelaide, Australia

**Keywords:** Endophthalmitis, Post-injection, anti-VEGF, *Morganella morganii*, *Enterococcus faecalis*, Vitrectomy

## Abstract

**Background:**

Endophthalmitis following intravitreal injection is a potentially devastating complication of anti-VEGF injections. Post-injection endophthalmitis due to *Enterococcus faecalis* is rare, and no previous case of *Morganella morganii* endophthalmitis after intravitreal injection has been reported.

**Case presentation:**

We present the first reported case of *Morganella morganii* and *Enterococcus faecalis* endophthalmitis after intravitreal injection in an immunocompetent patient in the absence of recent ocular surgery. Our patient presented with hand movement visual acuity one day after anti-VEGF injection and demonstrated no clinical improvement despite repeated intravitreal ceftazidime and vancomycin injections. A decision was made to proceed with early vitrectomy given failure of intravitreal antibiotics. Visual acuity improved to 6/90 at 12 weeks after vitrectomy without any evidence of disease recurrence.

**Conclusions:**

Post-injection endophthalmitis due to concurrent *Morganella morganii* and *Enterococcus faecalis* infections can have visually devastating consequences despite repeated empirical and targeted intravitreal antibiotics. Lack of clinical improvement following intravitreal antibiotics should warrant consideration of early vitrectomy. Our experience is a pertinent reminder of the ever-growing threat of uncommon and multi-resistant bacteria that must be considered when treating infections such as post-injection endophthalmitis.

## Background

Endophthalmitis following anti-VEGF therapy is an uncommon but recognized complication of intravitreal injections. Visual sequalae of endophthalmitis can be devastating despite early and targeted treatment. Coagulase-negative *Staphylococcus* is the most common pathogen isolated in post-injection endophthalmitis, although the list of pathogens is ever-increasing [1–3]

While previous cases of *Enterococcus faecalis* endophthalmitis, a gut commensal, [4] after anti-VEGF have been reported, endophthalmitis following intravitreal injection due to *Morganella morganii* has not. *Morganella morganii* is an unusual opportunistic pathogen, known for causing infections in patients with underlying comorbidities [5]. Neither organism is well-known for causing endophthalmitis.

Here we report a rare case of post-injection endophthalmitis secondary to concurrent *Enterococcus faecalis* and *Morganella morganii* with a better-than-expected final visual outcome.

## Case

A 75-year-old female presented to ophthalmic outpatient clinic for a routine aflibercept intravitreal injection into her left eye (LE) for choroidal neovascular membrane (CNVM). Best corrected visual acuity (BCVA) was 6/9 − 1 right eye (RE) and 6/9 − 2 LE. The following day she re-presented to ophthalmic emergency with a painful, injected LE, and significantly worse vision. BCVA was 6/9 − 1 RE and hand movement (HM) at 1 m in the LE, with an intraocular pressure (IOP) of 15mmHg RE and 31mmHg LE. Slit lamp examination demonstrated circumferential conjunctival injection, fine corneal keratic precipitates (KPs), a deep anterior chamber (AC) with 3 + cells, fibrin overlying the pupil, and posterior synechiae. No hypopyon was seen and posterior segment examination was obscured due to anterior segment inflammation. Right eye examination was unremarkable.

A diagnosis of LE post-injection endophthalmitis was made and the patient was treated with an intravitreal tap and injection of 0.1mL (2.25 mg) ceftazidime and 0.1mL (1 mg) vancomycin. She was commenced on 1-hourly prednisolone acetate 1% drops to the LE. She was immunocompetent with no significant past medical history and not on any regular medications. Ocular history included cataract surgery in both eyes performed more than ten years earlier.

On review the next day she reported ongoing LE discomfort and poor vision. Left eye BCVA was HM at 1 m and IOP was 34mmHg. Examination revealed persistent conjunctival injection, fibrin deposits on the corneal endothelium, and a deep AC with 4 + cells and early fibrin aggregation. A 1.8 mm hypopyon was noted. Posterior synechiae and fibrin deposits over the pupil were unchanged, and there was no red reflex or posterior segment view. Microscopy and culture of the previous day’s vitreous aspirate showed gram positive cocci; however, no classification or sensitives were available.

She was reviewed again the next day and BCVA in the LE had declined perception of light (PL) without projection, with the patient reporting increasing ocular discomfort. Conjunctival injection was persistent and dense stromal oedema with Descemet’s membrane folds (DMFs) were seen on corneal examination. The hypopyon had increased to 3.0 mm, and free-floating fibrin in the AC was obscuring the intraocular lens (IOL). Vitreous aspirate cultures now showed growth of *Enterococcus faecalis (E. faecalis)* and *Morganella morganii (M. morganii)*, and a decision was made to repeat intravitreal injection with the same doses of ceftazidime and vancomycin, and additional 0.1mL (0.4 mg) dexamethasone. She was also commenced on oral ciprofloxacin 750 mg twice daily (BD) and hydrocortisone acetate 1% ointment at night to the LE. Prednisolone acetate drops were reduced to 2-hourly to the LE.

**Fig. 1:**
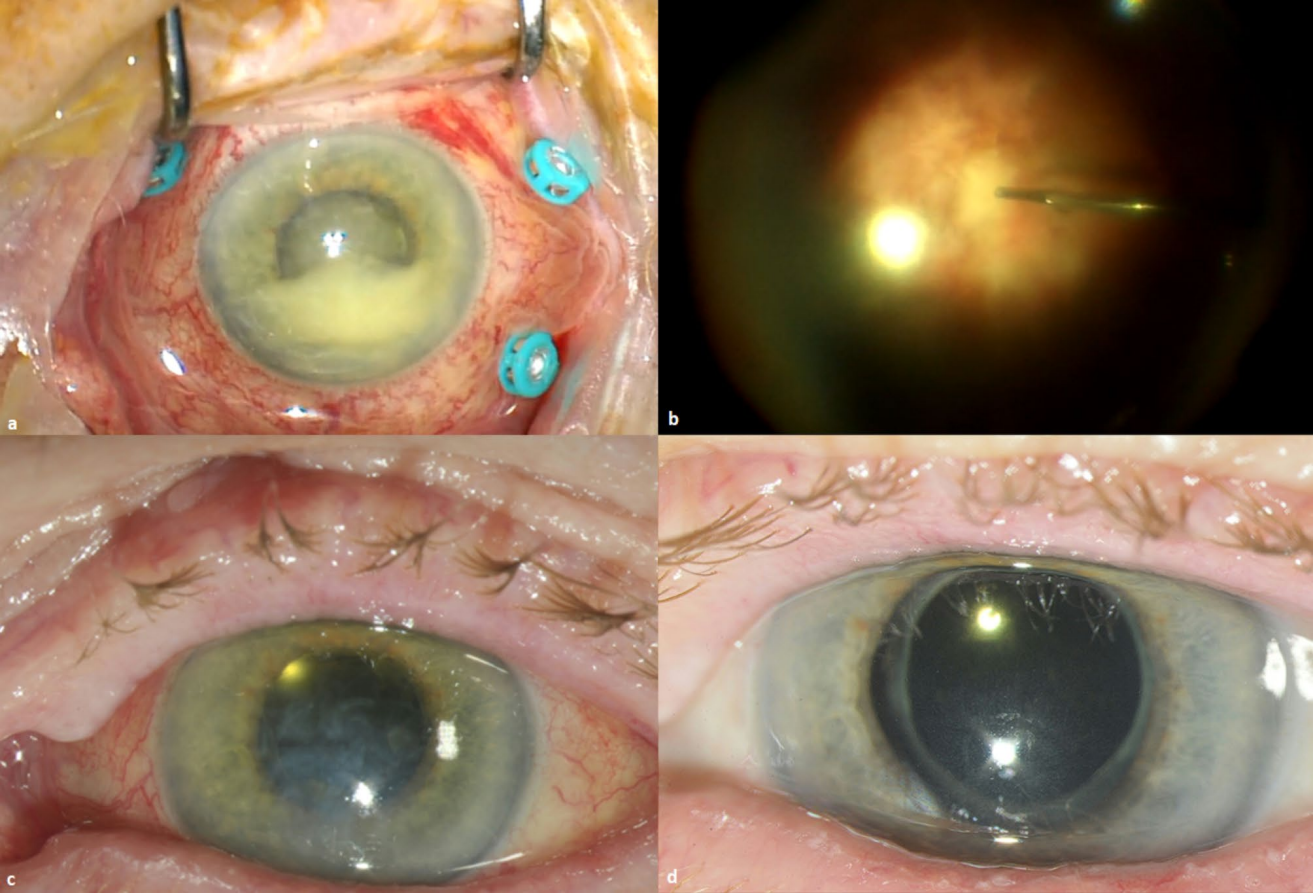
(a) Left eye at time of vitrectomy with hypopyon; (b) Fundus view during vitrectomy showing thick exudates; (c) One day after vitrectomy; (d) One week after vitrectomy with stable visual acuity and resolved pain

She was reviewed daily over the next two days without any improvement in LE BCVA, and had a persistent 3.0 mm hypopyon with no posterior segment view (Fig. 1.a). RE remained unremarkable. By this stage antimicrobial sensitivities revealed *E. faecalis* was sensitive to vancomycin and ampicillin, while *M. morganii* was sensitive to cefepime, ceftazidime, ceftriaxone, ciprofloxacin, gentamicin, piperacillin/tazobactam, and trimethoprim/sulfamethoxazole, and resistant to ampicillin and amoxicillin/clavulanate. Upon discussion with the patient a decision was made to proceed with pars plana vitrectomy (PPV), as there was no clinical improvement despite multiple intravitreal antibiotics.

Our patient subsequently underwent uncomplicated LE PPV with further intraoperative intravitreal vancomycin 1 mg, ceftazidime 2.25 mg, and dexamethasone 0.4 mg. Thick exudates filling the entire vitreous cavity were noted during surgery (Fig. 1. b). On day one post-PPV our patient had LE BCVA of PL with projection and IOP of 2mmHg (Fig. 1. c). Examination showed diffuse corneal oedema and DMFs, a deep AC with 2 + cells and fibrin, and a present red reflex without fundus view. Her LE pain had almost fully resolved. She was discharged home on day three post-PPV on LE 6 times daily chloramphenicol 0.5% minims and prednisolone sodium phosphate 0.5% minims, hydrocortisone acetate 1% ointment at night, oral ciprofloxacin 750 mg BD (10 days total), and a course of oral prednisolone 30 mg daily weaning by 5 mg each week (total six weeks). No recurrence of endophthalmitis was noted one week later (Fig. 1. d).

**Fig. 2:**
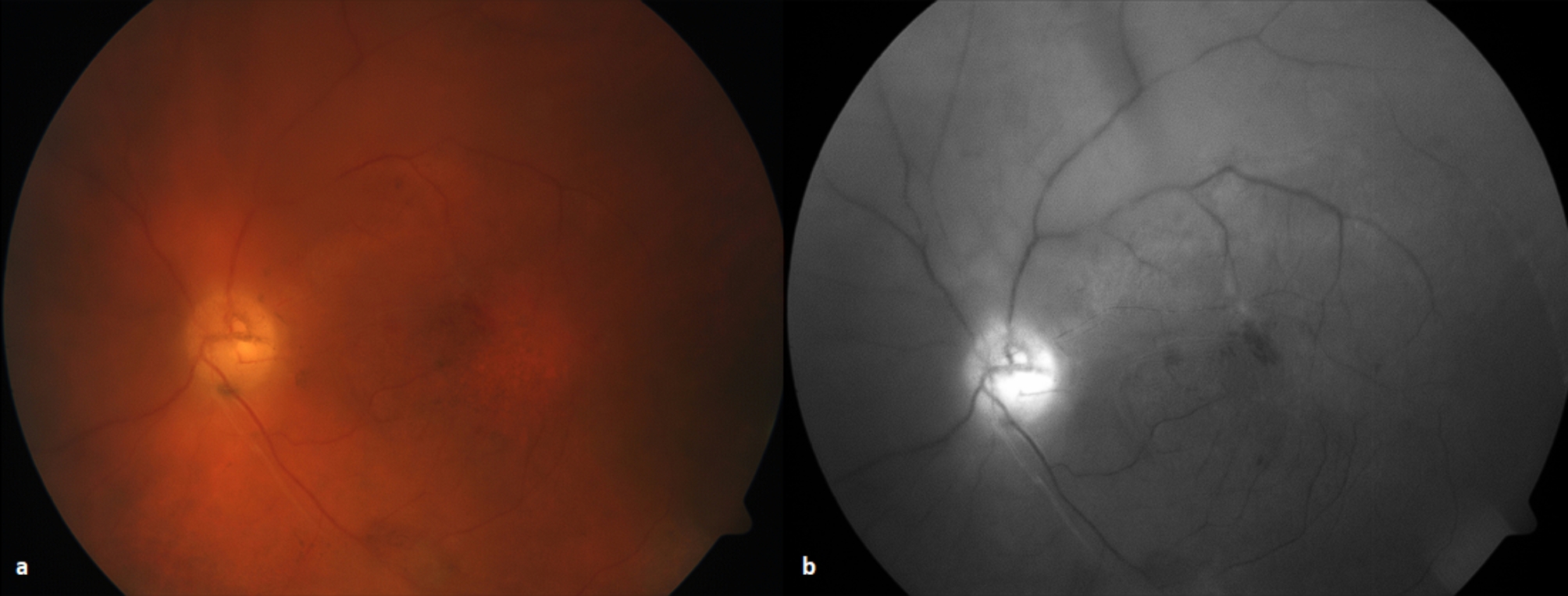
(a) Left eye color fundus photograph two weeks after vitrectomy; (b) Red-free fundus photograph at the same time

At follow up two weeks later, her LE BCVA had improved to 6/300. Examination demonstrated a clear vitreous with a small hemorrhage at the fovea, and an otherwise flat peripheral retina (Fig. 2). There were no signs of disease recurrence. Chloramphenicol was ceased, and topical prednisolone minims were reduced to BD for four more weeks before stopping.

At her last outpatient review at 12 weeks post-vitrectomy, her LE BCVA was 6/90 with an IOP of 16mmHg. Examination of the anterior and posterior segments was stable, without any disease recurrence.

## Discussion and conclusions

This unusual case of post-injection endophthalmitis was caused by *E. faecalis* and *M. morganii*. While *E. faecalis* is very rarely isolated in endophthalmitis following anti-VEGF, [6, 7] this is the first report of post-injection *M. morganii* endophthalmitis.

Acute endophthalmitis is an infrequent complication of intravitreal anti-VEGF injections. Post-injection etiology represents 8.5% of all endophthalmitis, while endophthalmitis following surgery accounts for 31.2% [2]. Coagulase negative *Staphylococcus* appears to be the most commonly isolated causative organism of post-injection endophthalmitis [1–3]. Reports of *M. morganii* endophthalmitis in the literature are very rare, and occur almost exclusively after ocular surgery [8–12]. A single occurrence of *M. morganii* endogenous endophthalmitis has been reported, [13] and no previous case has occurred following anti-VEGF injection. Sterile inflammation following intravitreal injection should also be considered; however, presence of pain, hypopyon, severe AC reaction, and significant vision loss can help distinguish an infectious from a non-infectious endophthalmitis [14]

*Enterococcus faecalis* is a gram-positive bacterium known to cause severe endophthalmitis and poor visual outcomes [15]. The pathogen is normally a gut commensal, however it is also known to cause many serious infections and exhibit resistance to multiple antibiotics [4] *E. faecalis* in our patient demonstrated an expected sensitivity to vancomycin, [15] which was thus an appropriate empiric agent. However, no improvement was seen after two doses of intravitreal vancomycin. This may have been due to concurrent *M. morganii* infection in our patient. *M. morganii* is a gram-negative, rod-shaped-bacillus of the enteric bacterium family and, along with *E. faecalis*, it can be found in the human gastrointestinal tract. It has been responsible for a range of infections in humans, most commonly urinary tract and post-operative wound infections [16, 17]. In recent years *M. morganii* has become more prevalent and increasingly difficult to treat due to its intrinsic and acquired accumulation of multidrug resistance genes [16]. Despite *M. morganii* having intrinsic resistance to third-generation cephalosporins, in our patient the pathogen was sensitive to ceftazidime – consistent with another recent case of endophthalmitis [9]. Resistance to ampicillin and amoxicillin/clavulanate was consistent with known resistance patterns [16]

First-line management of infectious endophthalmitis generally involves intravitreal tap and injection of antimicrobial agents. Our patient experienced no clinical improvement despite two intravitreal injections of ceftazidime and vancomycin. The decision to treat post-injection endophthalmitis with vitrectomy may depend upon severity of VA impairment and persistent vitritis following tap and inject [2]. Although evidence for early vitrectomy (within 48 h of presentation) is inconsistent, [18–20] its role in *M. morganii* endophthalmitis may be beneficial [9]

We report the first case of *M. morganii* endophthalmitis following intravitreal injection in an immunocompetent patient with no recent ocular surgery. Our patient developed symptoms of LE endophthalmitis 24 h after intravitreal aflibercept, associated with rapid deterioration in visual acuity. Early vitrectomy for *M. morganii* endophthalmitis may be indicated [9] especially if no improvement is seen within 48 h intravitreal antibiotics [19]. Although visual prognosis of *M. morganii* and *E. faecalis* endophthalmitis is poor, our patient’s visual outcome at final follow-up was better than expected [8, 10, 11]

## Data Availability

All data generated and analyzed during this study are included in this published article. Data and material are available from the corresponding author Michael Kvopka (E-mail address: michael.kvopka@gmail.com).

## Abbreviations

AC: Anterior chamber
BCVA: Best-corrected visual acuity
CNVM: Choroidal neovascular membrane
DMF: Descemet’s membrane fold
HM: Hand movements
IOL: Intraocular lens
IOP: Intraocular pressure
KP: Keratic precipitate
LE: Left eye
PL: Perception of light
PPV: Pars plana vitrectomy
RE: Right eye
VEGF: Vascular endothelial growth factor
